# Identification of biomarkers for tuberculosis susceptibility via integrated analysis of gene expression and longitudinal clinical data

**DOI:** 10.3389/fgene.2014.00240

**Published:** 2014-07-24

**Authors:** Qingyang Luo, Smriti Mehra, Nadia A. Golden, Deepak Kaushal, Michelle R. Lacey

**Affiliations:** ^1^Mathematics Department, Tulane UniversityNew Orleans, LA, USA; ^2^Division of Bacteriology and Parasitology, Tulane National Primate Research Center, Tulane UniversityCovington, LA, USA

**Keywords:** TB biomarkers, clinico-genomic models, integrated analysis, Bayesian hierarchical B-splines, TB susceptibility

## Abstract

Tuberculosis (TB) is an infectious disease caused by the bacteria *Mycobacterium tuberculosis* (*Mtb*) that affects millions of people worldwide. The majority of individuals who are exposed to *Mtb* develop latent infections, in which an immunological response to *Mtb* antigens is present but there is no clinical evidence of disease. Because currently available tests cannot differentiate latent individuals who are at low risk from those who are highly susceptible to developing active disease, there is considerable interest in the identification of diagnostic biomarkers that can predict reactivation of latent TB. We present results from our analysis of a controlled longitudinal experiment in which a group of rhesus macaques were exposed to a low dose of *Mtb* to study their progression to latent infection or active disease. Subsets of the animals were then euthanized at scheduled time points, and granulomas taken from their lungs were assayed for gene expression using microarrays. The clinical profiles associated with the animals following *Mtb* exposure revealed considerable variability, and we developed models for the disease trajectory for each subject using a Bayesian hierarchical B-spline approach. Disease severity estimates were derived from these fitted curves and included as covariates in linear models to identify genes significantly associated with disease progression. Our results demonstrate that the incorporation of clinical data increases the value of information extracted from the expression profiles and contributes to the identification of predictive biomarkers for TB susceptibility.

## 1. Introduction

Tuberculosis (TB) is an infectious disease caused by the bacteria *Mycobacterium tuberculosis* (*Mtb*) that affects millions of people worldwide. While a small fraction of individuals who are exposed to *Mtb* either completely clear the infection or develop active disease, the majority enter a state of latency, in which an immunological response to *Mtb* antigens is present (typically diagnosed by a positive response to a skin test) but there is no clinical evidence of infection. However, there is not a clear delineation between latent and active infection, with levels of bacterial activity encompassing a latency spectrum that varies considerably among individuals (Barry et al., 2009; Delogu et al., 2013). This considerably complicates the diagnosis and proactive treatment of TB infection, as currently available tests cannot differentiate individuals who are at low risk of disease progression from those who are highly susceptible to developing active disease. For this reason, there is much interest in the identification of diagnostic biomarkers that can predict reactivation of latent TB (Wallis et al., 2010; Walzl et al., 2011; Ottenhoff et al., 2012).

One approach for studying the progression of *Mtb* infection from initial exposure to latency is through controlled time-course experiments. Non-human primates (NHPs), such as rhesus or cynomolgus macaques, display a continuum of TB infection which cannot be reproduced in other animal models (Capuano et al., 2003; Dutta et al., 2010; Mehra et al., 2010, 2011, 2013) and are therefore optimally suited for this type of research. In experiments conducted at the Tulane National Primate Research Center (TNPRC), a set of 17 rhesus macaques were exposed to a low dose of *Mtb* bacteria and monitored for clinical signs of disease. Subsets of the animals were then euthanized at scheduled time points, and granulomas taken from their lungs were analyzed for gene expression using microarrays. While in an ideal setting the combined expression profiles produced from this experiment would provide a valuable snapshot into the processes by which latency is established at the genomic level, this would require that all of our subjects have a common experience of infection. In our study, a review of the clinical profiles associated with the animals revealed considerable variability in the nature of their respective illnesses, with some subjects exhibiting no clinical signs of infection and others presenting symptoms associated with severe disease. Based on this heterogeneity, we considered that an integrated analysis of our gene expression data that incorporated each animal's clinical history would provide greater insights into the genetic processes associated with the establishment of latent infection than one which considered only the length of exposure associated with each profile.

The term *clinico-genomic modeling* describes a large class of methods that seek to integrate data from high-throughput experiments with clinical information, typically for diagnostic or prognostic purposes (Gevaert et al., 2006). For example, a recent study of primary breast cancer recurrence demonstrated that the incorporation of traditional clinical risk factors improved disease outcome predictions over statistical classification tree models that only used genomic data (Pittman et al., 2004). However, to our knowledge ours is the first effort to incorporate longitudinal clinical data from a controlled experiment in the identification of genomic biomarkers from gene expression profiles.

We first incorporate a number of clinical covariates to model disease progression for each subject using a Bayesian hierarchical B-spline approach. Our methods are similar to those used in the development of longitudinal models for lung function in children observed in the presence of varying levels of ambient air pollution (Berhane and Molitor, 2008), although in our setting the response variable is an unobserved disease severity score rather than a measured quantity. From our estimated clinical trajectories, we can visualize and quantitatively compare the progression from exposure to latent infection in each of our subjects. We then incorporate disease severity estimates derived from these curves along with temporal information to identify genes that are significantly associated with either or both of these predictors. Our results demonstrate that the incorporation of clinical data significantly increases the amount of information extracted from the expression profiles and is consistent with other recent efforts to identify biomarkers for TB susceptibility.

## 2. Materials and methods

### 2.1. Experimental design

Seventeen Indian rhesus macaques were exposed to 100 cfu *Mtb* CDC1551 via *Mtb* aerosol, a dose which is known to induce latent TB based on prior research (Dutta et al., 2010). The animals were randomly assigned to time points at 2-week intervals for experimental euthanasia, at which point their lungs were biopsied and granuloma lesions were extracted. The experiment was conducted using previously established protocols (Dutta et al., 2010; Mehra et al., 2011), and all procedures were approved by the TNPRC Institutional Animal Care and Use Committee (IACUC) and the Tulane University Institutional Biosafety Committee (IBC). During the course of the post-exposure period, the subjects were routinely weighed and monitored for body temperature at regular intervals, with temperature reported as the change from a pre-exposure baseline (denoted by TMP) and weight reported as a percentage of the pre-exposure weight (denoted by WT). C-reactive protein (CRP), a known indicator of TB infection, was also monitored, and chest X-rays were taken at tri-weekly intervals and scored by a TNPRC radiologist on a 0–3 scale based on visual evidence of lesions (CXR). Gene expression profiles for extracted granulomas were assayed using Agilent TNPRC Macaca mulatta 4 x 44k Arrays (GPL10183) and scanned using the GenePix 4000B Scanner. All data files are available for download via the GEO Data Series GSE56919.

### 2.2. Hierarchical B-spline models

We use piecewise polynomials to estimate the trajectory of the unobserved disease progression *z*(*t*) as a linear combination of a set of B-Spline basis functions (de Boor, 1993). We restrict *z*(*t*) to the family of non-negative cubic polynomials on [0, τ_1_] and [τ_1_, τ_2_], where 0 < τ_1_ < τ_2_, with continuous second derivatives globally on [0, τ_2_]. Then *z*(*t*) can be expressed as *z*(*t*) = *c*_0_*B*_0,3_(*t*) + *c*_1_*B*_1,3_(*t*) + *c*_2_*B*_2,3_(*t*) + *c*_3_*B*_3,3_(*t*) + *c*_4_*B*_4,3_(*t*) where {*c*_0_, *c*_1_, *c*_2_, *c*_3_, *c*_4_} is a set of non-negative coefficients and {*B*_0,3_(*t*), *B*_1,3_(*t*), *B*_2,3_(*t*), *B*_3,3_(*t*), *B*_4,3_(*t*)} is the set of B spline basis functions. For each subject, τ_2_ is the duration of time from exposure to euthanasia and is known for each subject, while τ_1_ is an interior transition point (or “knot”) that is estimated. We use the notation *z*(*t*|**θ**) where **θ** = (*c*_0_, *c*_1_, *c*_2_, *c*_3_, *c*_4_, τ_1_, τ_2_).

The four variables WT, TMP, CRP, and CXR were measured longitudinally for the *n_s_* = 17 experimental subjects. To reduce the high degree of fluctuation in CRP measurements, we discretized the observations to fall into one of three ordered groups based on previous clinical observations: Level 1 for CRP = 0; Level 2 for 0<CRP<=10; and Level 3 for CRP >10.

Let *y*_*vil*_ be the value of the *v*th variable for the *i*th subject at the time point *t*_*vil*_, where *v* = 1, 2, 3, 4 (which correspond to WT, TMP, CRP, and CXR, respectively), *i* = 1, 2, · · ·, *n_s_* and *l* = 1, 2, · · ·, *n_vi_* denotes the time points observed for the corresponding subject and variable. At the initial time of *Mtb* exposure for each subject, we set ***y***_*i*0_ = {100, 0, 0, 0}, denoting the baseline state for each subject *i*. Let *Y* = *y_vil_* be the array of all observations for all subjects.

We define the following hierarchical model for the relationship between the observed clinical variables and unobserved disease state *z*(*t*|**θ**):

For *i* = 1, 2, …, *n_s_*:

(1) $$\begin{array}{l} y_{1il}=b_{10}+b_{11}z(t_{1il}|\theta_{i})+\epsilon_{1il}\\ \text{ for }l=1,\;2\cdots ,\;n_{1i} \end{array}$$

(2) $$\begin{array}{l} y_{2il}=b_{20}+b_{21}z(t_{2il}|\theta_{i})+\epsilon_{2il}\\ \text{ for }l=1,\;2,\cdots ,\;n_{2i} \end{array}$$

(3) $$\begin{array}{l} \text{logit}(P(y_{3il}\leq g))=\beta_{g}+b_{31}z(t_{3il}|\theta_{i})\\ \text{ for }l=1,\;2,\cdots ,\;n_{3i}\text{ and }g=0,\;1 \end{array}$$

(4) $$\begin{array}{l} \text{logit}(P(y_{4il}\leq h))=\alpha_{h}+b_{41}z(t_{4il}|\theta_{i})\\ \text{ for }l=1,\;2,\cdots ,\;n_{4i}\text{ and }h=0,\;1,\;2 \end{array}$$

Random errors ϵ_*vil*_ ~ *N*(0, σ^2^_*v*_), *v* = 1, 2 are independent for each subject and variable. Equations (3, 4) are proportional odds models (Agresti, 2002) for the discretized CRP *y*_3*il*_ ∈ {0, 1, 2} and the CXR *y*_4*il*_ ∈ {0, 1, 2, 3} in which logit$(x)=\frac{x}{1-x}$ and β_*g*_ and α_*h*_ are non-decreasing in *g* and *h*. The parameters *b_vj_* and σ^2^_*v*_ are common for all subjects while **θ**_*i*_ represents the effects specific to subject *i*, and we assume that the measurements *y*_*vil*_, *v* = 1, 2, 3, 4 are conditionally independent given *b_vj_*, σ^2^_*v*_, and **θ**_*i*_.

We note that the slopes *b*_*v*1_ and the disease states *z*(*t_vil_*|**θ**_*i*_) are not individually identifiable in our model since they only appear as a product. However, this is simply a matter of scaling and does not impact our ability to compare estimated disease trajectories across subjects.

#### 2.2.1. Parameter estimation

Let **θ**_*i*_ = (*c*_*i*0_,*c*_*i*1_,*c*_*i*2_,*c*_*i*3_,*c*_*i*4_, τ_*i*1_, τ_*i*2_) be the parameter vector for subject *i*. The disease progression function for the *i*th subject *z*_*i*_(*t*) is defined on the interval [0, τ_*i*2_], in which τ_*i*2_ is set to be the final time point, corresponding to the last measurement for any of the four clinical covariates. We assume that the pre-exposure disease state *z*(0|**θ**_*i*_) = 0, so we set *c*_*i*0_ = 0. Therefore, among the components of **θ**_*i*_, we need only estimate *c*_*i*1_,*c*_*i*2_,*c*_*i*3_,*c*_*i*4_, τ_*i*1_.

The intercepts *b*_10_, *B*_20_, β_0_, β_1_, α_0_, α_1_, α_2_ in Equations (1–4) determine the distribution of the corresponding clinical variables given the disease state is 0. Since initial values for WT and TMP were constant for all subjects at 100 and 0, respectively, the intercept terms *b*_10_, *b*_20_ were also fixed. Intercepts for the CRP and CXR probabilities at disease state 0 were also set to reflect the fact that pre-exposure measurements for these variables were fixed at 0 for all subjects. Values for the vector {β_0_, β_1_, α_0_, α_1_, α_2_} were set to be {5, 9, 5, 9, 13}, corresponding to the initial state probability $P(CRP=0)=P(CXP=0)=\frac{exp(\beta_{0})}{1+exp(\beta_{0})}=0.993$ (while these were somewhat arbitrary choices, we note that adjustments to these initial values were observed to have a negligible impact on the fitted models).

The remaining parameters are {***b***, **σ**^2^, **θ**_1_, · · ·, **θ**_*n_s_*_} where ***b*** = (*b*_11_, *b*_21_, *b*_31_, *b*_41_), **σ**^2^ = (σ^2^_1_, σ^2^_2_), and **θ**_*i*_ = (*c*_*i*1_, *c*_*i*2_, *c*_*i*3_, *c*_*i*4_, τ_*i*1_) for *i* = 1, 2, · · ·, _*n*_*s*__. Equations (1–4) specify that the WT and TMP values *y*_1*il*_ and *y*_2*il*_ are normally distributed while *y*_3*il*_ and *y*_4*il*_ have multinomial distributions. Using the conditional independence of the clinical variables, the joint likelihood function of the parameters *L*(***b***, **σ**^2^, **θ**_1_, · · ·, **θ**_*n_s_*_|***y***) is just the product of the marginal likelihoods.

We set prior distributions for these parameters to reflect our biological intuition and imposed boundary constraints to account for the lack of identifiability associated with our model. As we expect that increasing disease severity should be associated with weight loss, elevated body temperature, and/or higher levels of CRP and CXR, we use truncated diffuse normal priors on the slopes: *b*_11_ ~ *N*(0, 100)*I*_[−10,0]_, *b*_21_ ~ *N*(0, 100)*I*_[0,10]_, *b*_31_ ~ *N*(0, 100)*I*_[−10,0]_, *b*_41_ ~ *N*(0, 100)*I*_[−10,0]_, where *N*(μ_0_, ξ^2^_0_)*I*_[*a*,*b*]_ is the truncated normal distribution with mean μ_0_ and variance ξ^2^_0_ whose support is the interval [*a*, *b*]. These priors, which correspond to the scaled normal distribution with support on the interval between the mean and one standard deviation to its right or left, place higher weight on values close to 0 but are not highly restrictive. For the variance parameters, prior distributions were motivated by preliminary inspection of the observed variability in the data and bounds were set to constrain the estimates to a reasonable range of values: 1/σ^2^_1_ ~ *U*[0.001, 0.6], 1/σ^2^_2_ ~ *U*[1, 25]. Priors for the basis functions were defined by τ_*i*1_ ~ *U*[0, τ_*i*2_], and *c_ij_* ~ *N*(0, 10)*I*_[0,+∞)_ for *j* ∈ 1, 2, 3, 4.

We estimated the parameters by sampling from the posterior distribution using MCMC as implemented in WinBUGS (Lunn et al., 2000). After a burn-in period of 1000 iterations, we ran the MCMC algorithm for 15,000 iterations and thinned the chain by taking every third point to produce an effectively uncorrelated sample of *T* = 5000 points from the posterior distribution.

#### 2.2.2. Simulation study

To test our method's ability to accurately estimate the progression of a specified disease trajectory from observed clinical covariates, we constructed 4 hypothetical disease scenarios and then simulated associated observations for WT, TMP, CRP, and CXR.

Our simulations represented the following cases: (1) progression to severe illness; (2) asymptomatic infection; (3) recovery following acute illness, and (4) gradual progression to moderate illness. Given a specified disease trajectory, we generated 100 simulated datasets of weekly clinical observations according to Equations (1–4). At each time point, WT and TMP observations were randomly generated from the normal distributions determined by Equations (1, 2) and the categorical CRP and CXR observations were taken to be the level with the maximum probability determined by Equations (3, 4). We set the intercepts *b*_10_ = 100, *b*_20_ = 0, β_0_ = 5, β_1_ = 9, α_0_ = 5, α_1_ = 9, α_2_ = 13 as above. The slopes were set to be *b*_11_ = −2, *b*_21_ = 0.6, *b*_31_ = −3, *b*_41_ = −3.5 and the precisions were set to be 1/σ^2^_1_ = 0.11 and 1/σ^2^_2_ = 2. These parameters were picked so that the simulated values resembled the observed measurements in our TB experimental data.

For each simulated dataset, we sampled from the posterior distributions of the parameters (including slopes, precisions and subject-specific parameters *c*_*i*1_,*c*_*i*2_,*c*_*i*3_,*c*_*i*4_,τ_*i*1_) via MCMC. Due to the large number of samples in our study, we reduced our computational requirements to include a burn-in period of 50 iterations and then we retained every third sample of the following 1000 iterations. The empirical median of the posterior distribution for each parameter was chosen as its estimate, and these were then used to generate the estimated trajectories of the disease state for the four subjects.

### 2.3. Linear models for gene expression profiles

Following estimation of the disease trajectories for each subject, we employed linear models to detect genes that are significantly associated with disease features.

In this setting, we fit gene-wise models as described in Smyth (2004), in which the response variables were the normalized log expression value associated with gene *g* for each of the *i* subjects and the covariates were derived from the estimated trajectory *ẑ*_*i*_(*t*).

Our two-channel microarray data included expression levels of 44,449 gene probes for 17 subjects, arranged in a loop design that included other control samples. We analyzed the two channels individually, normalized the arrays using the Bioconductor packages LIMMA and OLIN (Smyth, 2005; Futschik, 2012), and extracted the data for the samples of interest.

To focus our attention on genes that would likely be associated with disease progression, we removed probes that showed insufficient variability across the subjects. Letting *u*_gi_ denote the *log*_2_ expression level for the *g*th gene probe of the *i*th subject, we used the following filtering criterion: exclude the *g*th gene probe from the analysis if (max_*i*_ *u_gi_* − min_*i*_ *u*_*gi*_) < 1.5.

For *J* clinical features *Z*_1_, *Z*_2_, · · ·, *Z_J_* as predictors of the expression profiles for the *G* probes remaining after the filtering step, we fit models of the form

(5) $$u_{g}=X\beta_{g}+e_{g}\text{ and }e_{g}\sim N(0,\;\sigma_{g}^{2}I)$$

for each *g* = 1, 2, · · ·, *G* where ***u**_g_* = (*u*_*g*1_, *u*_*g*2_, · · ·, *u_gS_*)^*T*^ ∈ ℝ^*S*^, the *S* × (*J* + 1) design matrix $X=\left(\begin{array}{ccccc} 1 & z_{11} & z_{21} & \cdots & z_{J1}\\ 1 & z_{12} & z_{22} & \cdots & z_{J2}\\ \vdots & \vdots & \vdots & \vdots & \vdots \\ 1 & z_{1S} & z_{2S} & \cdots & z_{JS} \end{array}\right)$ and **β**_*g*_ = (μ_*g*_, α_*g*1_, α_g2_, · · ·, α_*gJ*_)^*T*^. The intercept μ_*g*_ is the mean expression level for the *g*th probe across the subjects and α_*g*1_, α_*g*2_, · · ·, α_*gJ*_ are the slopes of the *J* clinical features for the *g*th probe.

We determined that a particular gene probe was significantly associated with the covariate combination *Z*_1_, *Z*_2_, · · ·, *Z_J_* if the *p*-values of the individual *t*-tests for the *J* covariates were all less than 0.05 and the *p*-value of the overall model *F* test *H*_0_: α_*g*1_ = α_*g*2_ = · · · α_*gJ*_ = 0 was less than 0.01. Given a set of competing models, the model with the smallest overall *p*-value for which all coefficients met the α = 0.05 significance level was selected as the best model for that probe.

Probe-level results were aggregated to the set of unique Agilent probe IDs. Because pairs of replicate probes occasionally were best fit by models with differing parameterizations, the statistical significance of the pooled coefficients was calculated by taking the geometric mean of the observed *p*-values (which were automatically assigned to the value 1 for any covariates that were not included in the best-fitting model for a given probe) and we reset any model coefficients that were not associated with aggregate *p*-values of 0.05 or less to be equal to 0.

Hierarchical clustering was performed in R to classify the unique probe IDs with respect to the scaled matrix of fitted model coefficients, using the Euclidean distance and Ward's minimum variance method.

### 2.4. Bioinformatics analysis

Agilent probe IDs that were significantly associated with a fitted clinical model were mapped to annotated genes or genomic regions using the DAVID Gene ID conversion tool (Huang et al., 2009a,b). Subsequently, the set of all unique mapped gene IDs was uploaded to the DAVID suite and analyzed for functional annotation. Our threshold for statistical significance of enriched terms was a Benjamini-Hochberg adjusted *p*-value of 0.05 or less.

The set of significantly enriched SP-PIR keywords was analyzed to determine whether representation of associated genes was equally distributed across the model-derived gene clusters. For each keyword, we conducted a Chi-Square goodness of fit test for the cluster distributions based on the null hypothesis of no association and calculated the associated *p*-values with adjustment for multiple testing using the Benjamini-Hochberg correction.

## 3. Results

Data for three of the subjects included in our study were found to be anomalous. In one case, terminal illness required immediate euthanasia, and in the two others we observed aberrant expression profiles suggesting experimental error. Data for these subjects were discarded and the results presented here correspond to the 14 remaining subjects.

### 3.1. Modeling of simulated trajectories

**Figure 1:**
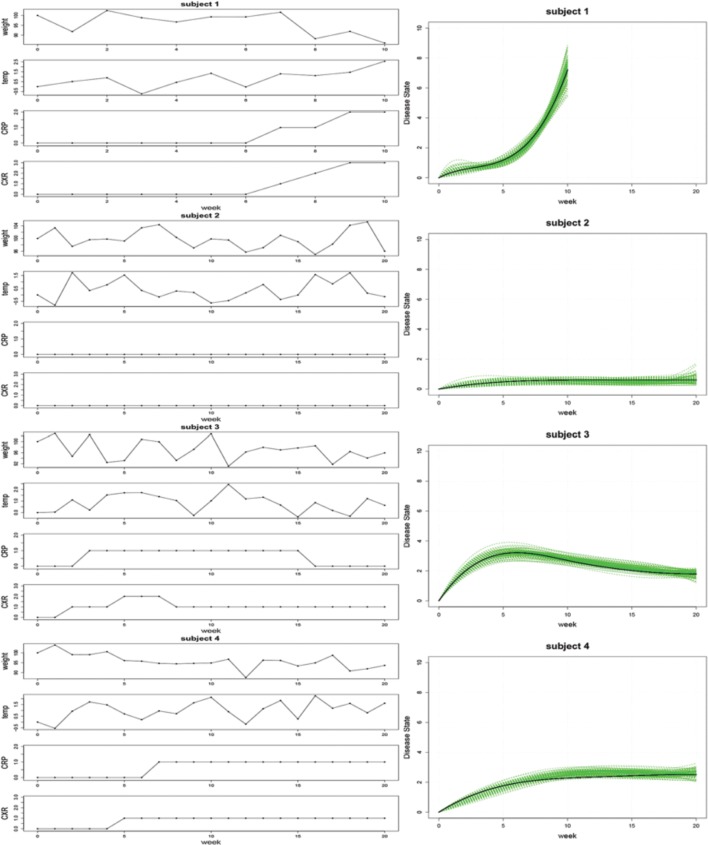
**Left:** An illustration of the simulated clinical observations for WT, TMP, CRP, and CXR associated with trajectories representing rapid disease progression (subject 1), minimal infection (subject 2), infection followed by recovery (subject 3), and gradually progressing infection (subject 4). **Right:** Estimated disease state trajectories for 100 simulated datasets (green curves) and the associated scaled true trajectory (black curve) for each of the four cases.

The results from our simulation study are shown in Figure 1. The 100 estimated trajectories for each of the four cases were compared to the true disease trajectory. As mentioned previously, the disease state is not identifiable and is unique only up to a constant. Therefore, the comparison was done in relative terms to see whether our modeling approach can recover the “shape” of the disease state trajectories. Specifically, suppose *z*_*i*_(*t*) is the true trajectory and *ẑ*_*i*_(*t*) is any one of the 100 estimated trajectories for the *i*th case where *i* = 1, 2, 3, 4. We expect that there exists a scaling constant γ such that γ *z*_*i*_(*t*) ≈ *ẑ*_*i*_(*t*) for all *i*, and the right panel of Figure 1 displays a plot of the 100 estimated trajectories *ẑ*_*i*_(*t*) against the scaled true trajectory γ *z*_*i*_(*t*). In each case, our simulations faithfully recovered the underlying trajectory, thereby demonstrating the ability of our approach to accurately estimate model parameters in practice.

### 3.2. Fitted trajectories for clinical profiles

**Figure 2:**
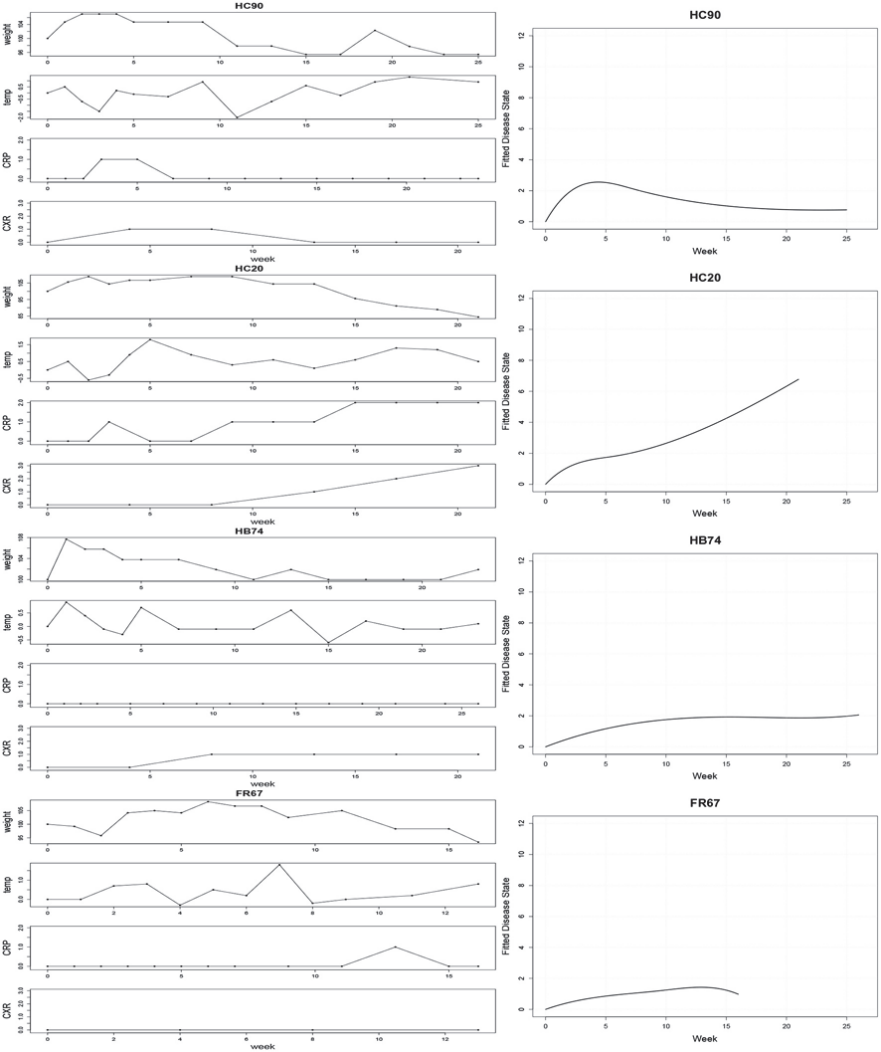
**Left:** Observed clinical variables for four typical subjects: HC90, HC20, HB74, FR67. CRP values are binned into 3 groups, corresponding to absent, low, or high CRP levels. **Right:** Estimated disease trajectories from Bayesian hierarchical B-Spline models incorporating the four clinical covariates.

**Figure 3:**
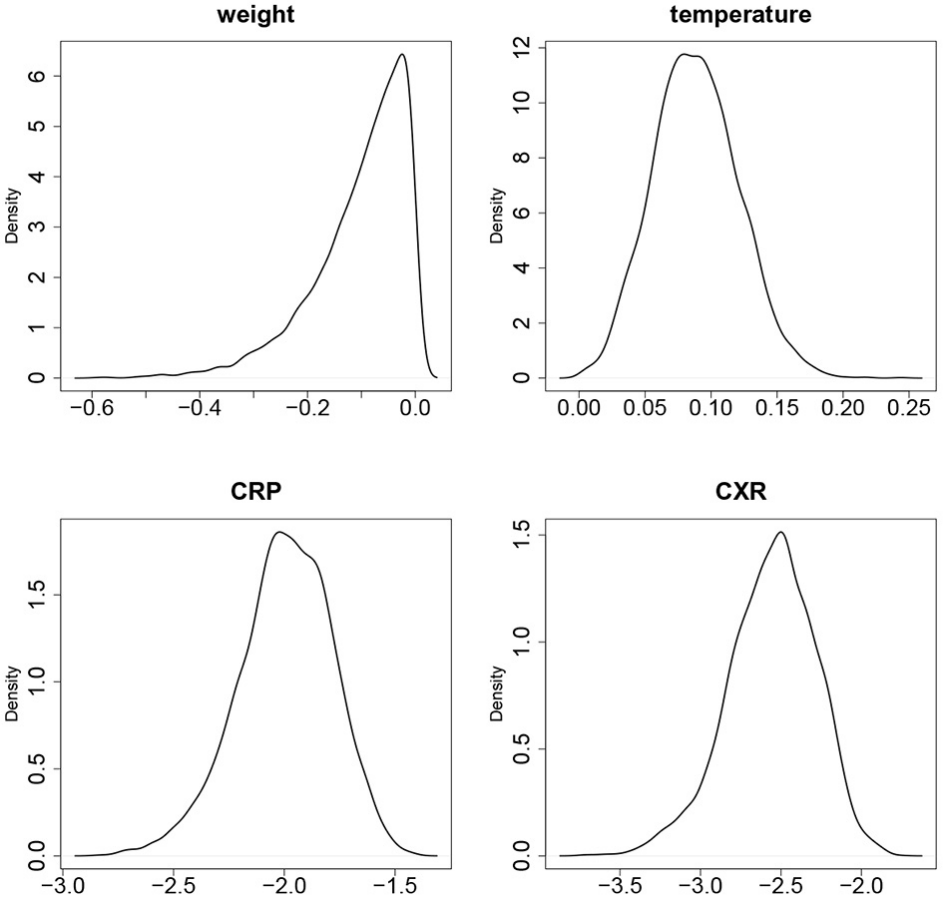
**The empirical densities of posterior distributions of the slopes *b*_11_ (WT), *b*_21_ (TMP), *b*_31_ (CRP), *b*_41_ (CXR)**.

The left panel of Figure 2 displays the observed clinical variables for four typical subjects: HC90, HC20, HB74, FR67. The CRP used in the plot is the discretized CRP. Hierarchical B-Spline models Equations (1–4) were fitted for each of the subjects as described. Following MCMC estimation, we obtained empirical posterior distributions for the common effects parameters, as shown in Figure 3 and summarized in Table 1.

**Table 1:** **Distributions of parameter estimates**.

**Parameter**	**Mean**	***SD***	**2.5%**	**Median**	**97.5%**
*b*_11_ (weight)	−0.1026	0.0874	−0.3228	−0.0804	−0.0032
*b*_21_ (temperature)	0.0885	0.0324	0.0285	0.0875	0.1549
*b*_31_ (CRP)	−1.9971	0.2141	−2.4630	−1.9860	−1.6160
*b*_41_ (CXR)	−2.5587	0.2746	−3.1631	−2.5420	−2.0809
1/σ^2^_1_ (weight)	0.0437	0.0043	0.0357	0.0436	0.0525
1/σ^2^_2_ (temperature)	1.3532	0.1358	1.0950	1.3465	1.6340

While all parameters were statistically significant, estimated trajectories, as shown in the right panel of Figure 2, were more sensitive to changes in CRP and CXR than to those in TMP and WT. Table 2 summarizes certain key features of the fitted trajectories for each subject. Time post-exposure *T* is defined by the time from initial exposure to euthanasia, final severity *S* is the value from the fitted disease trajectory at the final time point, onset time *O* is the time when the subject initially reaches a specified severity level (which here was taken as 1.4 based on estimated severity scores associated with symptomatic illness), and the maximum severity *M* is the highest value attained for a given trajectory.

**Table 2:** **Features of fitted disease trajectories**.

**Subject**	**Time post-exposure**	**Final severity**	**Onset time**	**Maximum severity**
BV21	15.0	2.95	8.1	2.97
CA75	15.1	1.96	12.0	1.96
FE10	23.0	2.20	5.0	2.20
FJ05	26.0	2.10	22.1	2.10
FR67	16.1	0.95	12.0	1.43
HA77	12.0	3.84	1.1	3.86
HB74	26.0	2.06	6.6	2.06
HC20	21.0	6.77	2.9	6.77
HC38	9.0	6.25	1.7	6.59
HC90	25.0	0.76	1.3	2.56
HG80	5.0	2.86	1.4	2.88
HJ01	21.0	3.91	3.7	4.04
HJ91	17.0	2.92	8.7	3.05
HJ93	9.0	4.51	2.9	4.51

### 3.3. Linear models for gene expression

After preliminary filtering, 14,504 candidate gene probes were retained for further investigation. Not surprisingly, preliminary analysis indicated that the predictors in Table 2 were highly correlated, and so we chose to focus our attention on two of the least correlated predictors, time post-exposure *T* and final severity *S*. For each of these probes, the best-fitting regression model was chosen from among models containing all subsets of linear and quadratic terms for *T* as well as a linear term for *S*. A total of 9130 probes were significantly associated with at least one of these models, and 8453 of these corresponded to annotated genes or genomic regions as identified by the DAVID Gene ID conversion tool. The probe level results were then aggregated to represent a set of 4864 unique Agilent probe IDs. As shown in Table 3, the great majority of genes (93%) were significantly associated only with post-exposure time *T*, with the remaining 7% associated with either severity *S* alone or with both *S* and *T*.

**Table 3:** **Best-fitting models for 4864 Unique Agilent Probe IDs**.

**Clinical covariates**	**Number of significantly associated genes**
*T_i_*	367
*T_i_*^2^	3959
*T_i_*, *T_i_*^2^	188
*S_i_*	145
*T_i_*, *S_i_*	25
*T_i_*^2^, *S_i_*	151
*T_i_*, *T_i_*^2^, *S_i_*	29

**Figure 4:**
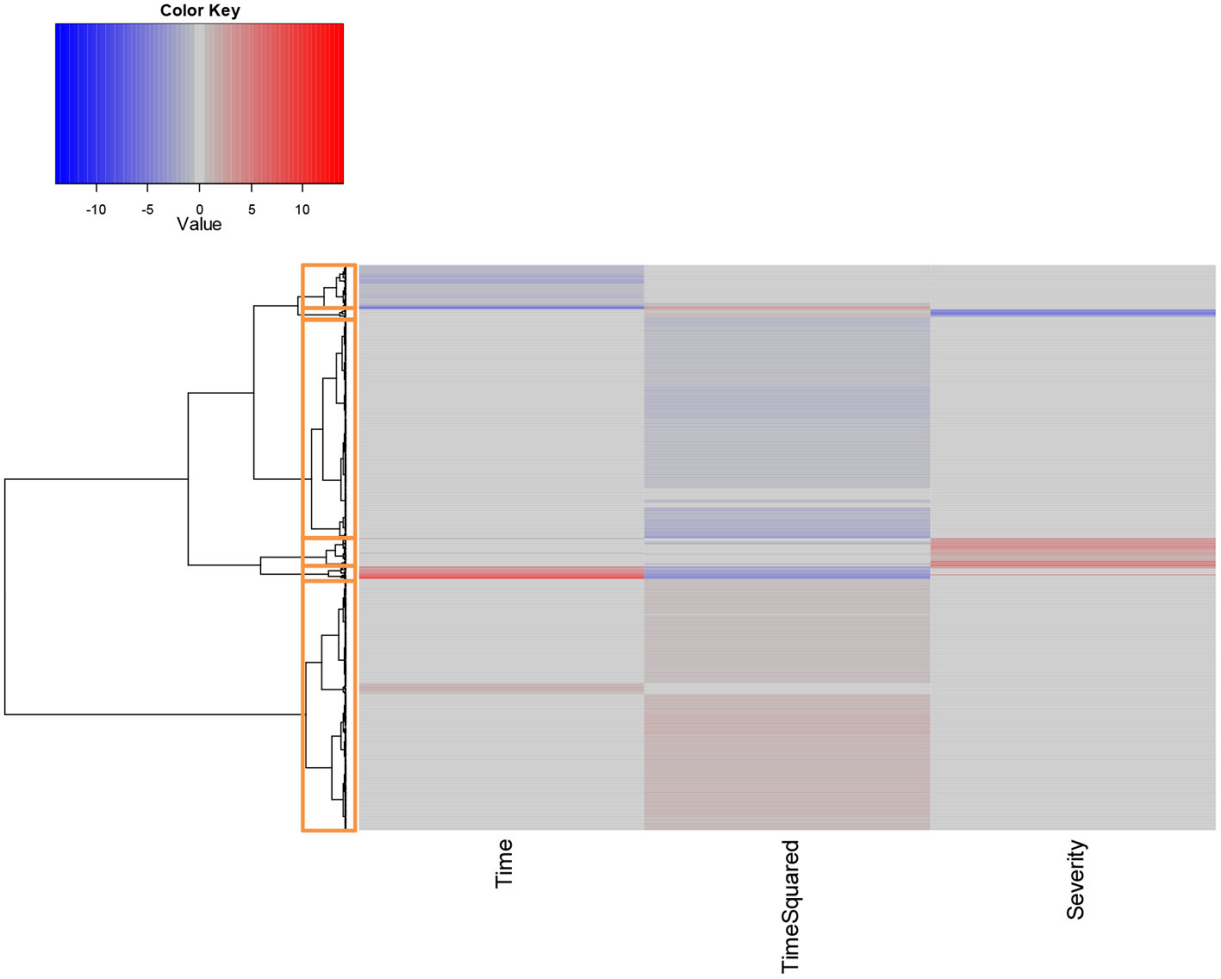
**Hierarchical clustering of unique probe IDs based on fitted model coefficients**. All coefficients were scaled prior to analysis to assign equal weight to each covariate. Red values denote coefficients that are positively associated with increases in the given covariates at the α = 0.05 significance level, while values in blue denote significant negatively associated coefficients.

Hierarchical clustering was performed on the scaled set of estimated model parameters to determine subsets of probe IDs that had the most similar characteristics with respect to their fitted models. Following visual inspection, we determined that 6 clusters were sufficient to adequately classify the results, as shown in Figure 4.

The largest cluster (which we denote Cluster 1) contained 2165 probes which mapped to 1950 distinct gene IDs. These expression profiles were predominantly characterized by quadratic increases in gene expression over time *T* (with linear increases in the remaining few). Cluster 2 contained 102 probes that displayed a parabolic trend, initially increasing and then decreasing in expression over time. These mapped to 100 distinct gene IDs. Cluster 3 (251 probes, 247 unique gene IDs) included probes whose expression significantly increased as a function of severity score *S*. Approximately half of these probes were also significantly associated with time *T*, either as a quadratic or linear term. Cluster 4 was the second largest (1891 probes, 1818 unique gene IDs) and exclusively included probes whose expression decreased as a quadratic function of time *T*. Cluster 5 was the smallest of the clusters, containing 74 probes mapping to 73 unique gene IDs. Expression profiles for probes in this cluster were all negatively associated with disease severity *S*, and the majority of these were also significantly associated with time *T*. Finally, Cluster 6 (381 probes, 373 gene IDs) included probes whose expression decreased linearly as a function of *T*.

### 3.4. Functional associations

**Figure 5:**
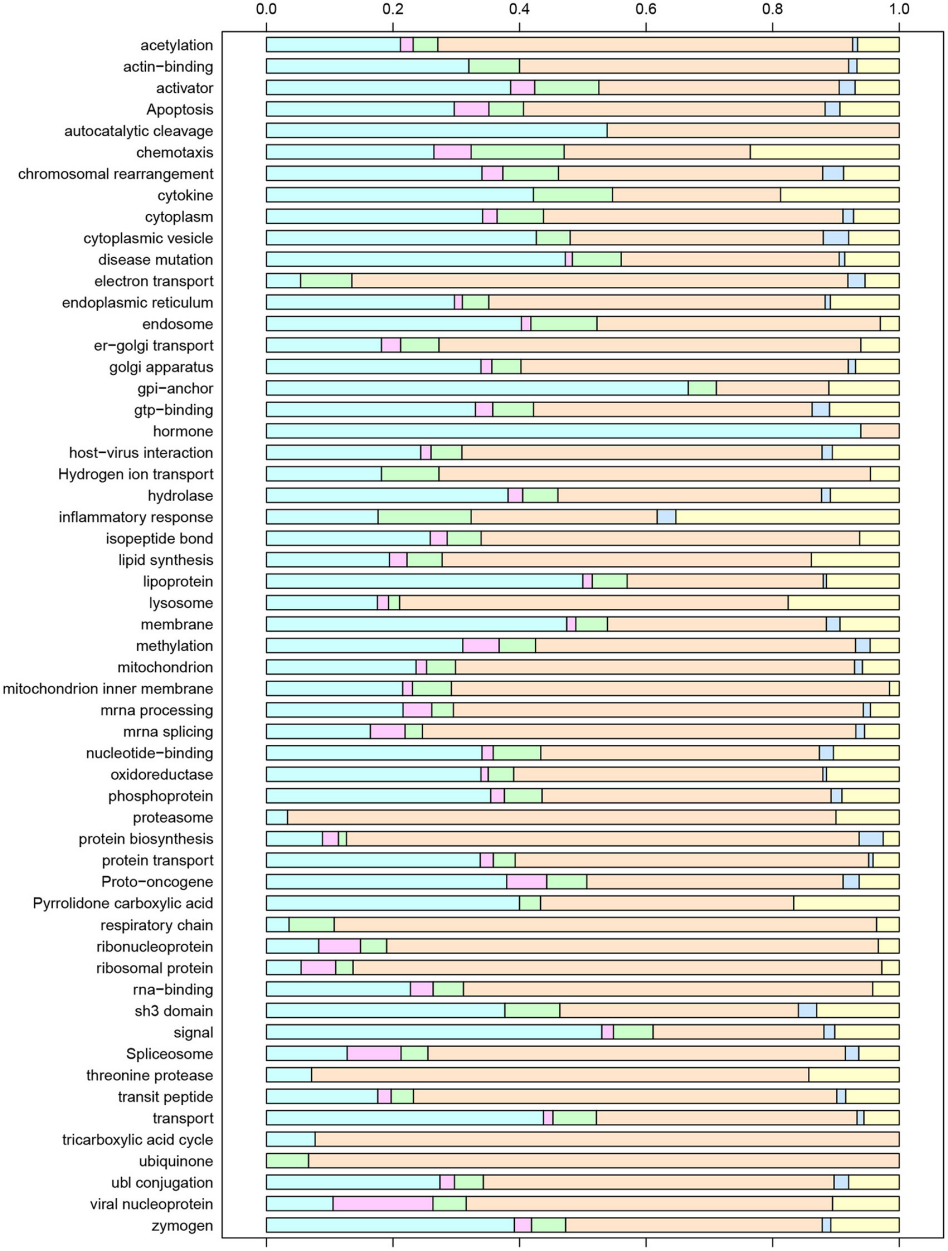
**Enriched SP-PIR keywords for the set of unique gene IDs associated with post-exposure time *T* and/or final severity score *S* at the α = 0.05 significance level**. For each keyword, the distribution of associated genes by model cluster is displayed using colored bars, with Cluster 1 on the left (light blue) and Cluster 6 on the right (yellow).

The set of all unique gene IDs included in the six clusters was imported into the DAVID bioinformatics tool suite and analyzed for functional annotation. We found that this subset was significantly enriched for 181 GO Biological Process (BP) terms, 50 Cellular Component (CC) terms, 7 GO Molecular Function (MF) terms, 8 KEGG Pathways, 77 Swiss-Prot Protein Information Resource (SP-PIR) Keywords, and 5 UniProt Sequence Annotation (UP-SEQ) Features. To avoid redundancy, we present results for the 56 statistically significant SP-PIR keywords that were unambiguously defined in the Swiss-Prot controlled vocabulary of keywords (www.uniprot.org/docs/keywlist). Figure 5 displays these terms along with the relative proportion of gene IDs included within each expression profile cluster. Based on the observed numbers of gene IDs in each cluster, if the gene IDs represented by a given keyword were randomly associated with the set of six clusters we would expect the percentage of gene IDs by cluster to be distributed as follows: 42.4% in Cluster 1, 2.2% in Cluster 2, 5.5% in Cluster 3, 39.7% in Cluster 4, 1.6% in Cluster 5, and 8.6% in Cluster 6. Chi-Square tests for random association of enriched genes with cluster membership identified 24 terms that were consistent with the expected cluster distribution, while the remaining 32 terms deviated significantly from the expected proportions. For a few terms, the deviations reflected an imbalance of gene IDs associated with quadratic temporal increases or decreases (Clusters 2 and 4), which would be expected to be observed in nearly equal proportions. For example, for the keyword “Hormone” 31 of 33 gene IDs were associated with Cluster 2 (*p* = 0.005) while 14 of the 15 gene IDs associated with the keyword “Ubiquinone” were contained in Cluster 4.

Of particular interest were the keywords that exhibited significant over-representation of genes associated with disease severity score, represented by Clusters 3 and 5. For the keyword inflammatory response, 6 of the 32 included gene IDs (17.6%) were severity-associated (CCL2, CCL11, CCL20, CXCL1, CXCL3, and TLR8), and over 10% of the gene IDs mapped to the keywords “activator,” “chemotaxis,” “chromosomal rearrangement,” “cytokine,” “electron transport,” “endosome,” and “SH3 domain” were included in Clusters 3 or 5. While relatively few genes were significantly down-regulated with increased severity, an interesting inclusion was the chemokine CXCR5. Recent studies have demonstrated that CXCR5 activity is essential for TB immune response (Gopal et al., 2013; Slight et al., 2013), and our results suggest that expression of this chemokine may be deficient in our most symptomatic subjects. CCL2, a chemokine which was positively associated with severity in our analysis, has also been proposed as a biomarker based on experimental findings demonstrating that expression of this gene was elevated for the more severe cases within a group of human patients (Hasan et al., 2009; Hussain et al., 2011; Ansari et al., 2013).

**Figure 6:**
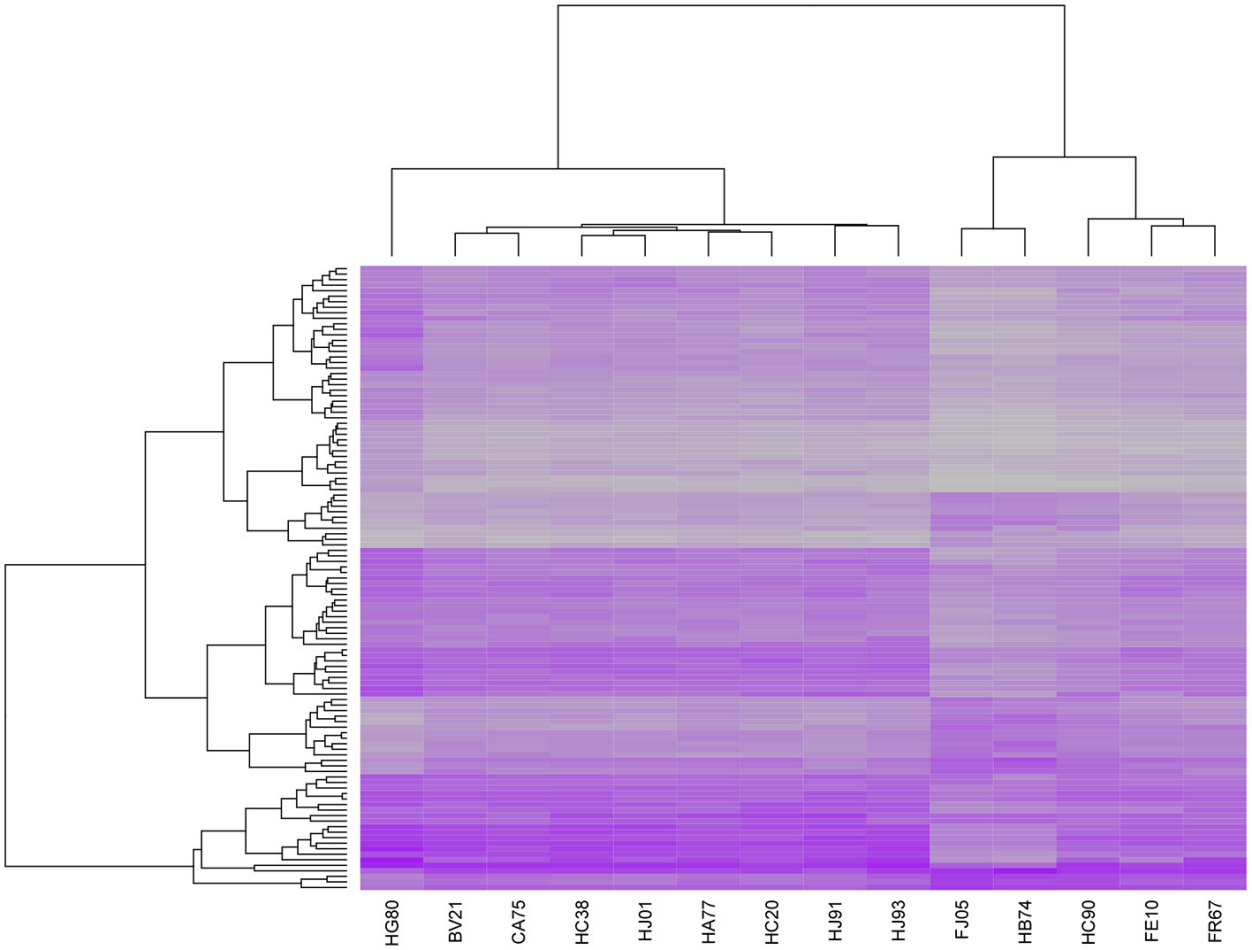
**Heatmap of expression profiles for 106 genes significantly associated with post-exposure time *T* and/or final severity score *S* and included in a set of 393 transcripts that were previously reported to effectively discriminate between cases of active and latent TB in blood samples (Berry et al., 2010)**. Hierarchical clustering of the samples on the basis of this subset separates 5 of the 6 least severe cases in our sample from the remaining 9 cases.

To put our results in a larger context, we compared our transcription profiles with those identified in recent studies seeking to identify TB expression biomarkers. In 2010, researchers published a list of 393 transcripts that were found to effectively discriminate between cases of active and latent TB in blood samples (Berry et al., 2010). We downloaded this list, which included 376 distinct gene IDs, and matched it to our set of 1950 genes that were significantly associated with *T* and/or *S*. We found an overlap of 106 genes and applied hierarchical clustering to the expression profiles associated with this subset. The results, shown in Figure 6, clearly separated five of our subjects from the others. Interestingly, these correspond to five of the six lowest scoring cases using our fitted severity models, all of which had very low CXR scores, 0 CRP values, and low or undetectable levels of bacteria upon autopsy. A very low-scoring subject on our severity scale that was not included in this group was CA75, an animal which demonstrated considerable weight loss despite an absence of other clinical symptoms and whose expression profile more closely resembled those of some of the more symptomatic subjects. Overall, we found that there was a significant difference in both time post-exposure and severity score between the subjects in the two clusters, although the temporal association may be due to the fact that the three animals that were studied for over 24 weeks were also coincidentally among the least symptomatic cases. We also performed a hierarchical clustering analysis on the set of profiles for 168 temporal- and/or severity associated genes included in a list of 409 gene IDs identified in a recent aggregate analysis of TB expression biomarkers identified in 7 prior studies (Joosten et al., 2013) and obtained identical clusters, suggesting that our results are fairly robust.

## 4. Discussion

The majority of clinico-genomic modeling efforts to date have emphasized the aggregation of clinical and genetic data in the prediction of binary disease outcomes. However, for infectious diseases such as TB that are associated with a spectrum of conditions, such an approach is unlikely to illuminate the subtle variations in genetic function that might predispose one individual to develop a more severe infection than another. As our experimental data clearly demonstrate, the progression from *Mtb* exposure to the development of latent infection is a far from uniform process. Even in controlled experiments such as ours, reactions vary considerably and the clinical response is difficult to predict. Therefore, the analysis of gene expression profiles to understand the development of latent infection will be of limited value unless such variation is taken into account.

Despite the obvious benefits of incorporating clinical data in this setting, little work has been done to facilitate such analyses by effectively aggregating a set of disparate longitudinal clinical measurements in a practical and intuitive way. To contribute to this important area of research, we have applied Bayesian hierarchical B-Spline models to the estimation of disease trajectories. Our fitted estimates provide helpful summaries of the clinical profiles of each of our subjects and enable the direct incorporation of aspects of the individual disease progressions in a quantitative form. Furthermore, the modeling of continuous disease trajectories offers a great deal of analytical flexibility. While in our particular study we concluded that the duration of time post-exposure and the estimated severity of disease at the time of euthanasia were the most important explanatory factors for variation in gene expression, one might imagine that in other settings different aspects of an estimated disease trajectory would be more predictive. For example, the frequency of bouts of acute illness might be more relevant for some conditions, while for others the time to recovery might be of particular interest.

As an alternative to predicting disease outcomes, we have focused our attention on the incorporation of clinical profiles in the identification of biomarkers associated with observed disease severity. Our results demonstrate that, even with a fairly limited set of subjects, our approach can identify key genes that have been shown to be factors in TB prognosis. This illustrates the potential of such integrated analyses for not only TB, but for a variety of complicated diseases in which subjects are monitored over time. While controlled experiments such as ours are, of course, limited to the laboratory setting, the ability to incorporate longitudinal clinical profiles in the analysis of gene expression data from human subjects is certainly an option in many observational studies and clinical trials. The estimation of individual disease trajectories in such studies would not only enable significant improvements in both the sensitivity and specificity of biomarker identification beyond current approaches, but would also provide insights into personalized treatment strategies.

## Author contributions

Qingyang Luo developed and implemented the models, analyzed the clinical and gene expression data, and contributed to the manuscript. Smriti Mehra infected the animals and collected samples. Nadia A. Golden produced the microarray data. Deepak Kaushal directed the primate experiments and contributed to the manuscript. Michelle R. Lacey directed the modeling project, performed the bioinformatics analysis, and revised and finalized the manuscript.

### Conflict of interest statement

The authors declare that the research was conducted in the absence of any commercial or financial relationships that could be construed as a potential conflict of interest.
